# Varespladib (LY315920) Appears to Be a Potent, Broad-Spectrum, Inhibitor of Snake Venom Phospholipase A2 and a Possible Pre-Referral Treatment for Envenomation

**DOI:** 10.3390/toxins8090248

**Published:** 2016-08-25

**Authors:** Matthew Lewin, Stephen Samuel, Janie Merkel, Philip Bickler

**Affiliations:** 1 Research and Development, Ophirex, Inc., Corte Madera, CA 94925, USA; 2 Center for Exploration and Travel Health, California Academy of Sciences, San Francisco, CA 94118, USA; 3 General Medicine, Queen Elizabeth Hospital, King’s Lynn, Norfolk PE30 4ET, UK; paulshania@yahoo.co.uk; 4 Yale Center for Molecular Discovery, Yale University, West Haven, CT 06516, USA; janie.merkel@yale.edu; 5 Anesthesia and Perioperative Care, University of California, San Francisco, CA 94143, USA; bicklerp@anesthesia.ucsf.edu

**Keywords:** snakebite, field treatment, varespladib, LY315920, methyl-varespladib, LY333013, inhibitor, envenomation, pre-referral, antidote

## Abstract

Snakebite remains a neglected medical problem of the developing world with up to 125,000 deaths each year despite more than a century of calls to improve snakebite prevention and care. An estimated 75% of fatalities from snakebite occur outside the hospital setting. Because phospholipase A2 (PLA2) activity is an important component of venom toxicity, we sought candidate PLA2 inhibitors by directly testing drugs. Surprisingly, varespladib and its orally bioavailable prodrug, methyl-varespladib showed high-level secretory PLA2 (sPLA2) inhibition at nanomolar and picomolar concentrations against 28 medically important snake venoms from six continents. In vivo proof-of-concept studies with varespladib had striking survival benefit against lethal doses of *Micrurus fulvius* and *Vipera berus* venom, and suppressed venom-induced sPLA2 activity in rats challenged with 100% lethal doses of *M. fulvius* venom. Rapid development and deployment of a broad-spectrum PLA2 inhibitor alone or in combination with other small molecule inhibitors of snake toxins (e.g., metalloproteases) could fill the critical therapeutic gap spanning pre-referral and hospital setting. Lower barriers for clinical testing of safety tested, repurposed small molecule therapeutics are a potentially economical and effective path forward to fill the pre-referral gap in the setting of snakebite.

## 1. Introduction

It has been estimated that more than 75% of deaths by snakebite occur outside the hospital setting and antivenom administration is limited to the hospital setting and cannot reverse damage already caused by venom toxicity at the time of presentation to antivenom capable facilities [1]. There is a clear, unmet need for effective and economical snakebite therapies that can be given rapidly and at the time of bite [2,3]. Versatile antivenom snakebite therapies have proven elusive since the outer structures of venomous molecules are highly variable and are known to present a difficult and inefficient target for antivenom [4,5,6,7]. Even more elusive has been the development of pharmacological snakebite therapies with either specific or broad-spectrum efficacy for snakebite envenomation and they are largely neglected from policy and drug pipeline reviews [2,8]. Inhibitor-based approaches to the treatment of snakebite have been attempted, but no broad-spectrum inhibitors of several key venom components common to all venomous snakes have been identified. Acetylcholinesterase inhibitors such as neostigmine and edrophonium have been administered intravenously for management of snakebite [9,10,11,12] yet their use in neurotoxic snakebite remains controversial [13]. Snake venom metalloproteinases (svMP) and serine protease (SP) inhibitors [2,14,15,16] have also been proposed with phospholipase A2 inhibitors as potential therapies, but potent, clinically plausible, broad-spectrum examples have not yet been identified with certainty [17,18,19,20,21] and remain elusive [22,23,24,25,26]. Snake venom PLA2s, in particular, play a critical role in early morbidity and mortality from snakebite, causing death by paralysis as well as destruction of tissues and derangement of homeostatic mechanisms critical for regulation of coagulation and oxygen transport [27,28,29,30]. Unless given shortly after a bite, antivenom is generally considered ineffective in the setting of neurotoxin-induced pathology because of its inability to penetrate peripheral and central nervous system tissues [13,31]. Additionally, snake venom sPLA2s are not as antigenic as larger, more foreign proteins, such as svMPs [22,23,24,25,26]. Thus, snake venom sPLA2s are as an ideal target for other types of therapeutics, such as small molecule therapeutics [2].

In this exploratory study, we describe repurposed (but never FDA approved) broad-spectrum, high potency snake venom sPLA2 inhibitors effective against medically important snake venom PLA2 enzymes from six continents. This broad-spectrum activity against snake venom is particularly surprising in view of our observation that varespladib and methyl-varespladib are not potent inhibitors of bee venom PLA2 and typically potency of medicinal PLA2 inhibitors is less for snake venoms than for mammals. Methyl-varespladib, a rapidly absorbed, orally bioavailable prodrug of varespladib [32] could be administered orally (e.g., as an elixir) so that a person with no or very limited skill could potentially initiate treatment outside a hospital setting. Methyl-varespladib is metabolized to varespladib, so the parent compound was the focus of our proof-of-concept studies though field use of an IV formulation is an unlikely scenario. The use of 3-substituted indoles for snake venom sPLA2 inhibition represents a possible springboard for the genesis of effective field treatments for snakebites; either could be rapidly developed with programmatic support and industry cooperation. Our findings warrant further investigation into the efficacy of veraspladib and methyl-varespladib in an even wider diversity of snakes to determine if either could be an essential component of the long sought-after venom antagonistic, first-line field-treatment for snakebite.

## 2. Results

### 2.1. Inhibition of sPLA2 Activities in Vitro

**Figure 1:**
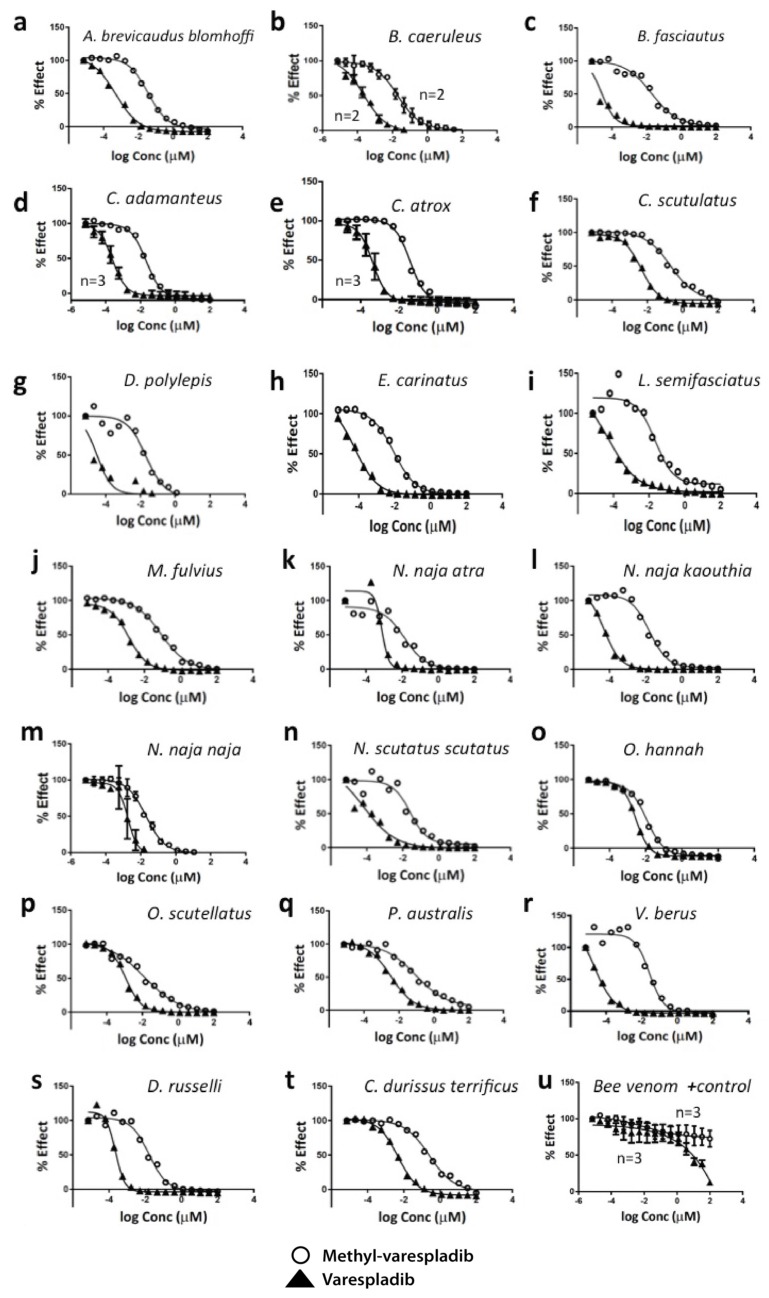
In vitro dose-response curves for varespladib and its orally bioavailable prodrug methyl-varespladib tested against 20 medically important snake venoms. While demonstrating high degrees of potency against snake venoms, neither varespladib nor methyl-varespladib showed high degrees of potency against bee venom sPLA2 (positive control). *N* = 1 run unless otherwise specified number of replicates. Error bars signify s.d. **a**. *Agkistrodon brevicaudus blomhoffi*, **b**. *Bungarus caeruleus*, **c**. *B. fasciatus*, **d**. *Crotalus adamanteus*, **e**. *Crotalus atrox*, **f**. *Crotalus scutulatus*, **g**. *Dendroaspis polylepis*, **h**. *Echis carinatus*, **i**. *Laticauda semifasciata*, **j**. *Micrurus fulvius*, **k**. *Naja naja atra*, **l**. *Naja naja kaouthia*, **m**. *Naja naja naja*, **n**. *Notechis scutatus scutatus*, **o**. *Ophiophagus hannah*, **p**. *Oxyuranus scutellatus*, **q**. *Pseudechis australis*, **r**. *Vipera berus*, **s**. *Daboia russelli*, **t**. *Crotalus durissus terrificus*, **u**. Bee venom (*Apis mellifera*) purified sPLA2 positive (+) control.

In examining drugs that could be repurposed for snakebite, we found that varespladib (LY315920) and methyl-varespladib (LY333013) inhibited the sPLA2 activity of large arrays of snake venoms in vitro using chromogenic assays, but did not show great activity against bee venom used as a standard, positive control (Table 1 and Figure 1). Additionally surprising was the observation that the IC_50_ of varespladib and methyl-varespladib for essentially all snake venoms tested was significantly lower than values ever reported for inhibition of mammalian, including human, sPLA2 [32].

### 2.2. Mouse in Vivo Pilot Experiments

#### 2.2.1. Pretreatment with Varespladib in an Elapid Envenomation Model

**Figure 2:**
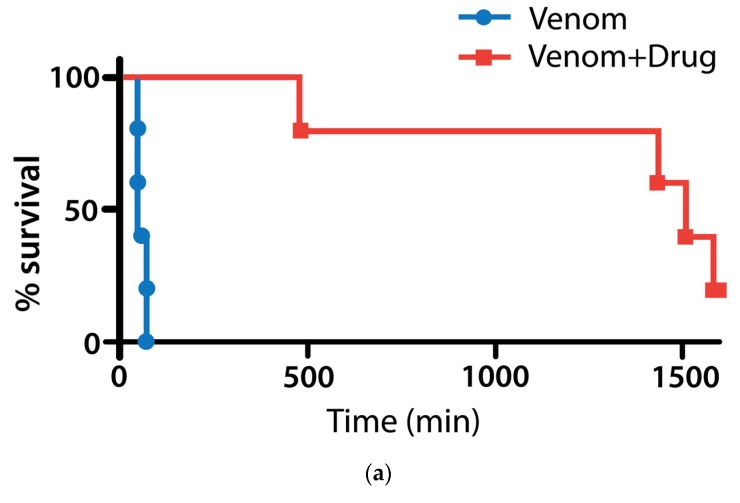

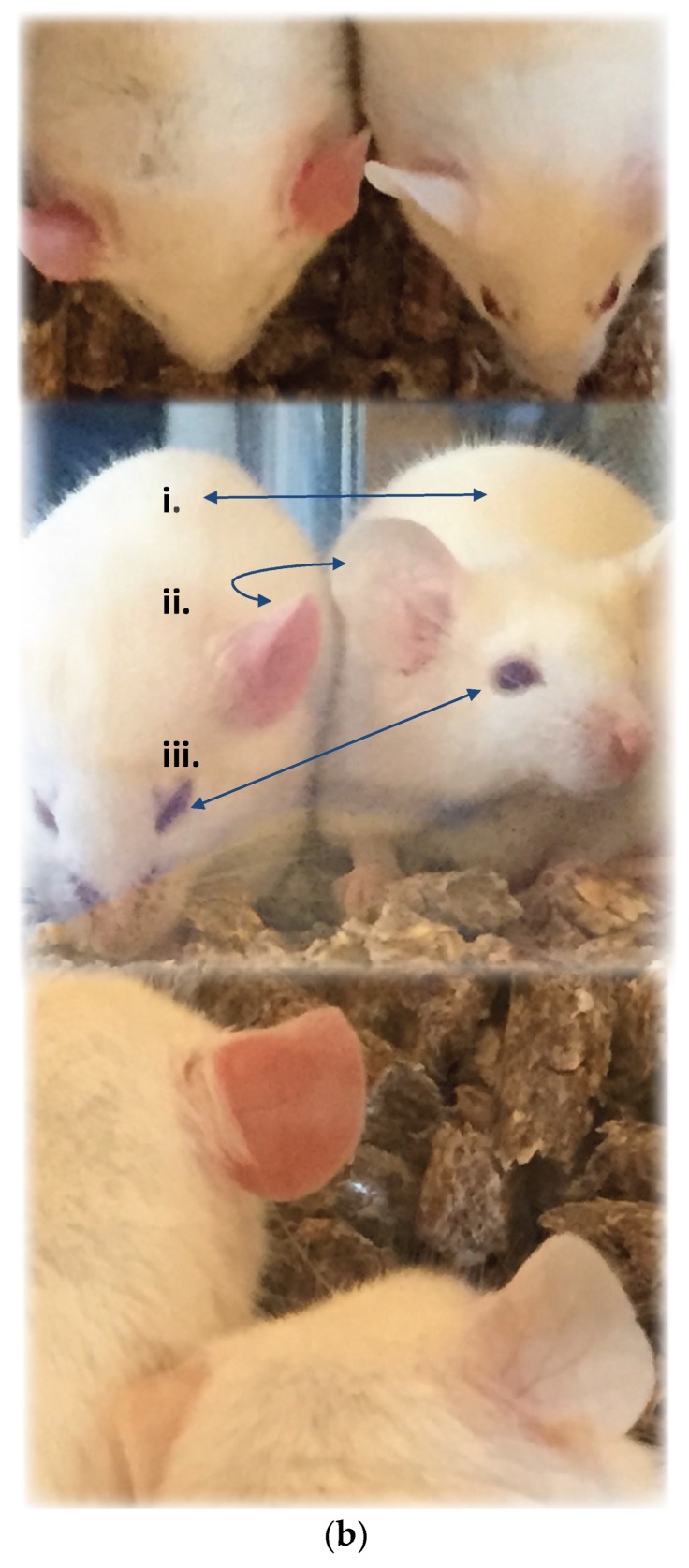
Pretreatment with varespladib protects against *M. fulvius* envenomation. (**a**) Five of 5 (100%) of mice given 4 mg/kg SC injections of *M. fulvius* venom died quickly with previously described paralytic and hemorrhagic complications. Zero of 5 (0%) of mice pre-treated with varespladib (4 mg/kg) several minutes before venom injection died within 8 h; (**b**) from a different experiment with methyl-varespladib, but exemplary of coral snake bite syndrome and effect of the study treatments: Left, untreated mouse 2 h after venom administration showing effects of venom including (i) postural weakness; (ii) vasodilation (ears) and (iii) ptosis; Right, methyl-varespladib treated mouse. Both mice have piloerection.

Based on their surprising in vitro anti-sPLA2 activity (Figure 1 and Table 1) we pilot tested the survival effect of varespladib in a mouse model of lethal snake envenomation. Eastern coral snake (*Micrurus fulvius*) has well-characterized venom causing both hemo- and neurotoxic effects in normal prey and human victims [33,34,35] and had the most potent sPLA2 activity in vitro (Table 1 and Figure 1j), thus it was chosen for the first experiments. Mice receiving subcutaneous injections of *M. fulvius* venom at ~4 times the expected LD50 (0.1 mg *M. fulvius* venom/animal for approximate dose of ~4 mg/kg) survived when pretreated with 4 mg/kg varespladib subcutaneously while 0 of 5 (0%) of mice pre-treated with varespladib (4 mg/kg) died within 8 h. The 5 (100%) of sham treated envenomed mice died at an average of 63 min, compared to 1140 min for varespladib treatment group (Figure 2a). Only one varespladib-treated mouse showed any evidence of hemorrhage on necropsy, but this was significantly less than the controls. The remaining mice showed no overt evidence of coagulopathy or hemorrhage at death.

The effects of varespladib wore off after approximately 24 h (1440 min) in 2 mice who died at very nearly 24 h with flaccid paralysis, but no apparent coagulopathic effects of the venom. One treated mouse died at 8 h post envenomation and had some signs of hemorrhage, but not in the lungs. Control mice died in a very close time period averaging 63 min (*p* < 0.0001 compared to varespladib treated mice, 1140 min). Two mice survived 30 h, both with persistent, but decreasing ptosis. Mice were only treated once in these experiments and dose finding and repeat dosing studies are needed for better characterization. No coagulation studies or histology were performed.

#### 2.2.2. Coinjection and Rescue against *Vipera berus* Venom

*V. berus* is one of the most widely distributed vipers in the world, ranging across Europe and Eurasia and as far north as the Arctic circle. It elaborates both hemo- and neurotoxins dangerous especially to children, pets and large animals such as horses [36,37,38,39,40,41,42].

**Figure 3:**
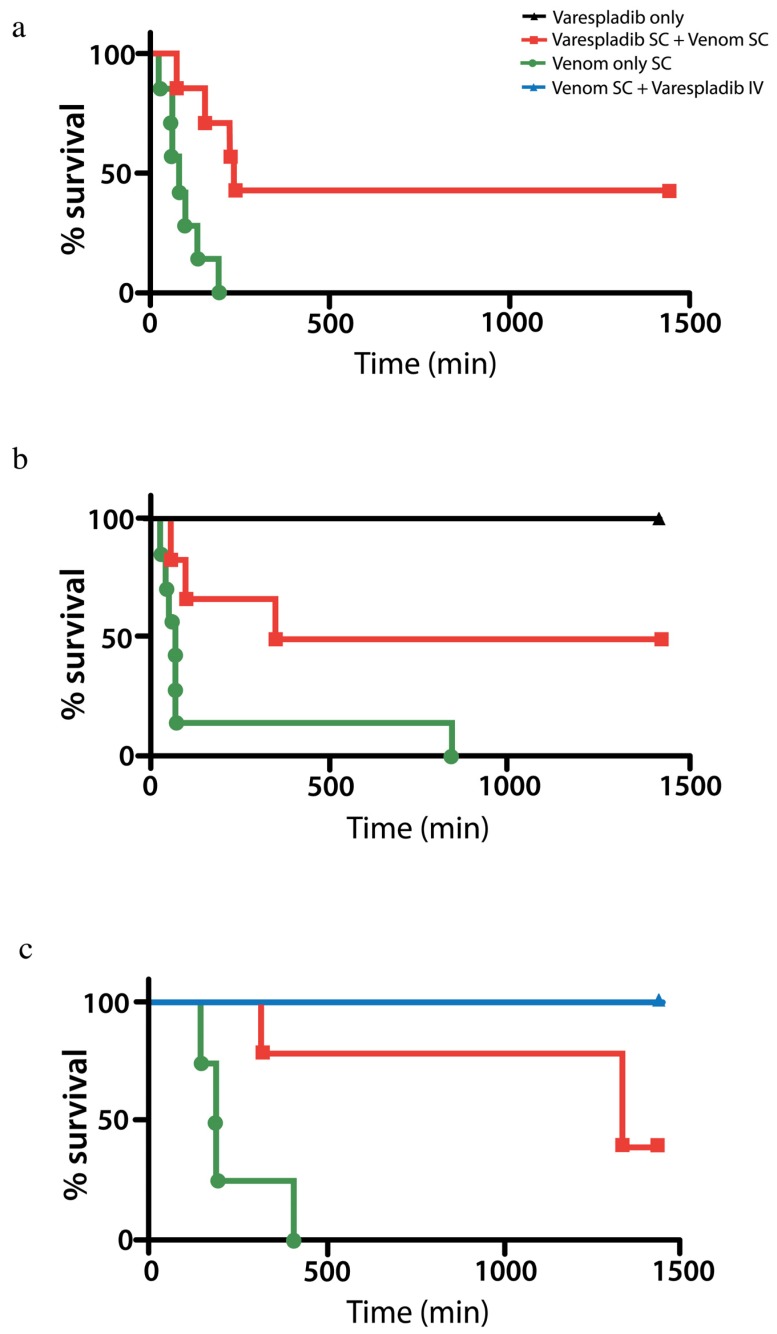
In vivo protection and rescue of *V. berus* envenomed mice by varespladib. (**a**) Venom and varespladib injected simultaneously into the subcutaneous space outlived controls (venom + excipient) *N* = 7 each group; (**b**) mice injected with lethal doses of venom just prior to SC administration of varespladib outlived controls (*N* = 7 each). Those injected with varespladib alone showed no signs of toxicity (*N* = 2); (**c**) varespladib administered SC or IV at a contract laboratory using the same lots of venom (8 mg/kg SC) and drug (8 mg/kg IV in lateral tail vein) following venom administration resulted in significant survival benefit with 5 of 5 IV treated animals surviving 24 h. 5 of 5 mice treated with SC varespladib outlived excipient only treated controls (*N* = 5 each group Survival: Controls 237 ± 92 min; Treated 1440 min. *p* < 0.001).

In pilot studies, mice injected with 100% lethal doses of *Vipera berus* venom outlived or were completely protected for 24 h from death when treated with varespladib administered subcutaneously (4 mg/kg unless stated otherwise) at the same time as or after venom administration (Figure 3a,b). All mice treated with IV varespladib following administration of venom survived 24 h, even when varespladib was administered after envenomation (Figure 3c). Mice injected with varespladib subcutaneously (SC) or intravenously (IV) alone showed no signs of toxicity. Venom only mice had subcutaneous hemorrhage, progressive paralysis and appeared to die from respiratory arrest. Treated mice had a similar, but unquantified degree of subcutaneous hemorrhage and initially had similar symptoms to controls for several hours before rallying such that it was difficult to distinguish control from treated animals with all appearing ill, initially after envenomation and up to roughly three h.

### 2.3. Rat in Vivo Modeling

**Figure 4:**
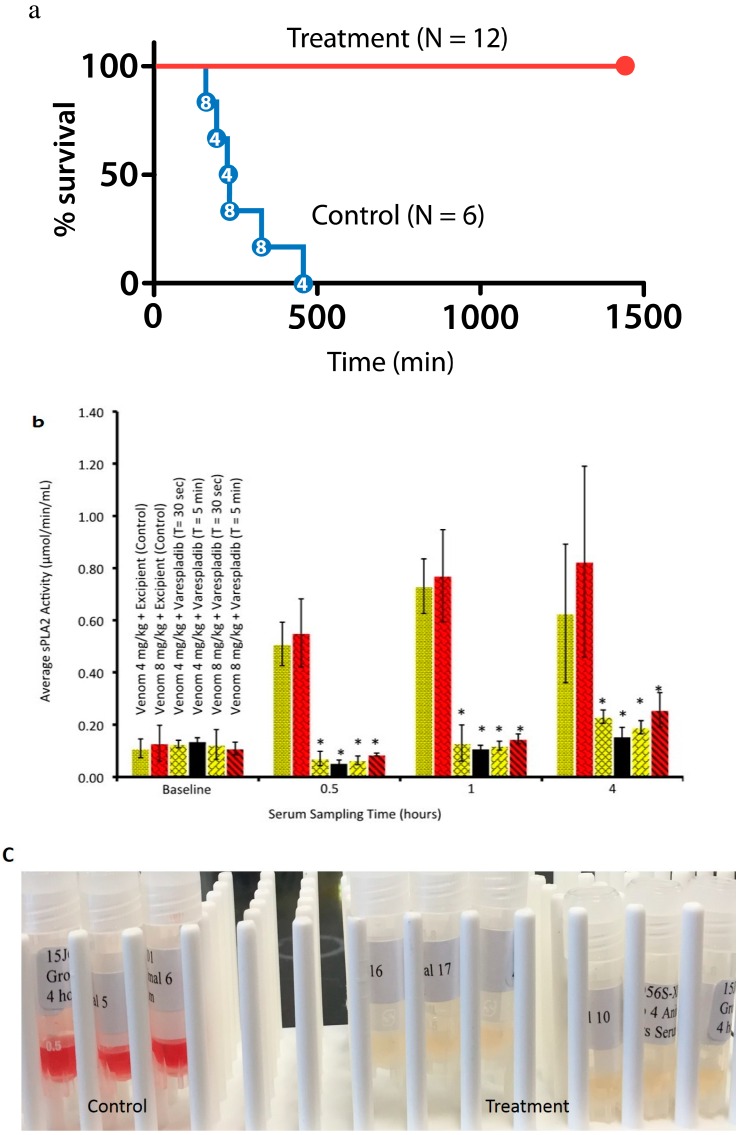
Rats given lethal doses of *M. fulvius* venom subcutaneously (SC) were treated with a single dose of Varespladib 8 mg/kg intravenously (IV) or excipient (control). (**a**) Plot showing survival benefit (Excipient *n* = 6, Varespladib *n* = 12). Dose of venom indicated inside the red dots indicate 4 mg/kg and 8 mg/kg venom doses for which there was no apparent difference in time to death (287 ± 110 min vs. 240 ± 68, respectively; *p* = 0.32); (**b**) snake venom-induced rise in sPLA2 activity is suppressed by varespladib (*N* = 3 each group; * *p* < 0.001); time of varespladib addition post venom-addition is noted by “T =” for each group; (**c**) treatment with varespladib prevented intravascular hemolysis in the same 12 animals. Tubes shown are blood drawn at the 4 h time point.

We also tested the survival effect of varespladib in a rat model of lethal snake envenomation. Rats dosed with either 4 mg/kg or 8 mg/kg of *M. fulvius* venom were entirely rescued by intravenous administration of varespladib within 5 min of venom injection (Figure 4a). Varespladib suppressed the venom-induced rise in sPLA2 activity of both doses of venom (Figure 4b), as well as venom hemolysis (Figure 4c).

## 3. Discussion

Our work presents a step toward the development of an affordable, broad-spectrum, first line antidote to snakebite using a repurposed small molecule inhibitor-based approach. Both varespladib and methyl-varespladib exhibit potent snake venom PLA2 inhibition in all species that have been tested so far and varespladib prevented the loss of life at 24 h in animal models selected for proof-of-concept studies and replicated in different laboratories. While the initial IC_50_ (in vitro) and proof-of-concept (in vivo) results are encouraging, further testing against more snake venom types is necessary before the drug can be considered a truly ‘agnostic’ inhibitor of enzymatic snake venom PLA2s and candidate broad-spectrum, single agent initial treatment for snakebite. Reproduction of our findings in multiple labs (Table 1, Figure 1, Figure 3c and Figure 4a) suggests the reliability of our original, small scale findings. Unreported herein, we have also found extended survival against *C. atrox*, *C. scutulatus* and *D. russelli* in small, as yet unpublished, pilot studies [43]. Particularly desirable in the near future will be systematic testing of these antidotes alone or in combination with repurposed metalloprotease inhibitors (e.g., prinomastat) in animal models against important snakes, such as those in the genus *Echis* [17]. A significant effect of varespladib against these venoms might further suggest suppression of the host’s reaction to the venom rather than direct inhibition of the *Echis* venom itself. We have no direct evidence for this in our studies to date however, others have recently observed this type of effect in mouse models of *Echis carinatus* envenomation [44]. Nevertheless, our results suggest varespladib-based therapies could be plausible first-line treatments for a diversity of snakebites alone or in combination with metalloprotease, serine protease and other inhibitors in the pre-referral setting. Most encouraging is the efficacy of varespladib when given during or even after envenomation (Figure 3 and Figure 4). While pretreatment of the venom or animal is an accepted practice in the process of testing for snakebite treatments, it does not reflect the reality of envenomation therapy, as victims generally do not have the foresight to pretreat themselves in the event of an envenomation. That said, a limitation of this work was the rapid IV administration of the therapeutics. Thus, our current conclusions are limited to the setting of rapid parenteral administration and its reliable bioavailability. Future studies should examine the effects of delays in varespladib administration using venom doses that more accurately reflect lethal human envenomation. A strength of our study was the high degree of lethality of the venom administration to controls and the separation of injection sites: Subcutaneous injection of venom at the scruff of neck and IV varespladib at the lateral tail vein using clinically plausible treatment doses (8 mg/kg) [45].

While these 1H-indole-3-glyoxylamides and related PLA2 inhibitors have failed as treatments for several chronic and acute conditions (e.g., rheumatoid arthritis, sepsis and acute coronary syndromes) in late-phase clinical trials [45,46,47,48,49] they have a good short-term safety profile [32,45,46,47] and to our knowledge have never previously been considered for treatment of snakebite [2]. Our data suggest dosing within ranges already tested in clinical trials for other indications [32,45] are likely in therapeutic range for snakebite treatment. Partnership with academia, other frameworks of social cooperation and industry already in possession of valuable pre-clinical and toxicological data, especially for off-patent compounds could speed development of these and other potential therapeutics for saving lives. Similarly, if these field-treatments can reduce healthcare costs related to snakebite even small reductions in the need for ICU and operative care, which even with no reduction in antivenom use, would be tremendously cost saving [50,51].

Unanswered questions include ideal dosing schedules and how best to develop these antidotes in programmatic fashion in conjunction with current standard therapy. Particular attention should be paid to efficacy of varespladib and methyl-varespladib through different administration routes, as methyl-varespladib is bioavailable after oral ingestion and offers a different therapeutic strategy than varespladib. For example, varespladib is immediately bioavailable by IV infusion, but methyl-varespladib can be formulated in a high-dose elixir with fast absorption in both the fed and fasted states [45]. Beneficial serum levels could be reached long before any intravenous therapy (i.e., antivenom) could be administered [45].

Varespladib and methyl-varespladib might prove to be sufficient in many situations to delay the effects of major envenomation—buying time for victims of snakebite—but, in the long-term, likely would need to be combined with other agents for more broad-spectrum, comprehensive treatment or serve as a bridge to survival to receive standard therapy. Snake venoms are comprised of many different toxin families, each of which can have independent effects on morbidity and mortality [52]. The fact that varespladib and methyl-varespladib can suppress host response safely and are more potent against snake venom PLA2 (lower IC_50_) than against mammalian sPLA2 may account in part for protection against the harmful effects of hemolysis, hemorrhage and other tissue destruction, but these effects need to be confirmed in more detailed studies not within the scope of this report. For example, we do not know exactly why we observed in every *Micrurus* study we performed, prevention of hemolysis and hemorrhage. Hemorrhage will occur if there is disruption of the clotting system, platelet dysfunction or break in the vascular integrity while hemolysis is the destruction of red cells that results in anemia. However, hemolysis and hemorrhage can occur via very different processes. Nevertheless, both processes have known direct and indirect interactions with PLA2 in *Micrurus* venoms. PLA2s have, additionally, involvement in hemolysis and hemorrhage related to the complement system in the setting of sepsis and vasodilatory anaphylotoxins [53] in some *Micrurus* (e.g., Figure 2b) and cobra venoms [54,55,56,57,58,59,60]. In addition to widening the diversity of snake venoms tested, future experiments would likely benefit from in-depth venomic analyses for the venoms to be tested, including second-generation venomics [61,62,63]. Ultimately, however, clinical testing where antivenoms are available for clinical study and comparison are necessary and are the only way to answer the critical questions of safety and efficacy in the setting of snakebite [2].

Varespladib and methyl-varespladib fit the profile of at least one key ingredient in hypothetical formulations to fill the pre-referral gap that has not been previously identified [2]. Development and dissemination of a rapidly absorbed, inexpensive and heat stable therapeutic would have immediate benefit for the health of people at risk of suffering life and limb-threatening snakebites. Aggressive investigation and development of field treatments for snakebite should not be stalled despite persistent lack of programmatic commitment by major global health organizations [2,8].

## 4. Conclusions

There is an urgent need for stable, economical and effective snakebite treatments that can be administered in the field or in rural areas where medical access is limited [2]. As both varespladib and methyl-varespladib have been extensively tested in Phase II clinical trials for unrelated indications, these compounds could be rapidly and economically evaluated as an initial treatment for snakebite [45,49]. This could be done alone and in combination with other small molecule therapeutics [2]. Given that the highest prevalence of snakebite mortality occurs in the pre-referral setting, the development of an orally bioavailable antidote could result in more people surviving and seeking referral for definitive antivenom therapy while IV formulations could be hospital based or used by skilled practitioners in a field setting. The best and most ethical means by which to do this needs to be scrutinized and carefully planned to avoid uncertainty about efficacy [64]. Nevertheless, the safety profile of these compounds and their potential to mitigate some of the early, lethally toxic aspects of snakebite envenomation make them reasonable candidates for consideration of such trials and warrant further examination by skilled practitioners and basic researchers in the field of snakebite.

## 5. Materials and Methods

### 5.1. In Vitro Experiments

Experiments were performed to assess sPLA2 activity using the 1,2-dithio analog of diheptanoyl phosphatidylcholine. sPLA2 catalyzes the hydrolysis of phospholipids at the sn-2 position yielding a free fatty acid and a lysophospholipid. The release of arachidonic acid from membrane phospholipids by PLA is believed to be a key step in the control of eicosanoid production within the cell. The Bee Venom PLA2 Control was a 100 μg/mL solution of bee venom PLA2 was supplied as a positive control from kits (Abcam kit catalog number ab133089). Assay optimization, screening and dose response measurements were performed at the Yale Center for Molecular Discovery. Experiments were performed in an assay buffer containing 25 mM Tris-HCl, pH 7.5 (Cayman Chemical, Ann Arbor, MI, USA), 10 mM CaCl_2_ (J. T. Baker), 100 mM KCl (Sigma, St. Louis, MO, USA), 0.3% Triton X-100 (Fluka) and 454 µM DTNB (Cayman Chemical) and plated into clear, Non-Treated 384-well plates (Corning, Corning, NY, USA). Venoms (Miami Serpentarium, Punta Gorda, FL, USA, and Sigma) were reconstituted in 1× phosphate-buffered saline (Lonza, Basel, Switzerland) to a concentration of 10,000 µg/mL. Crude, unfractionated lyophilized venom purchased from Sigma (*E. carinatus* and *D. russelli*) or the Miami Serpentarium (all others) was used in all cases. Varespladib and methyl-varespladib were purchased from Chemietek (Indianapolis, IN, USA) and dissolved in DMSO for in vitro experiments and bicarbonate/dextrose for in vivo experiments. The activity of venoms with 0.375 mM 1,2-bis(heptanoyl) Glycerophosphocholine (Cayman Chemical), the sPLA2 substrate, was selected based on kinetic enzymatic assays conducted at room temperature. Concentrations of venom was selected for screening and potency studies in which high sPLA2 activity was observed relative to any background activity of no venom control wells, and for which there was negligible substrate depletion at 60 min. The range in final concentrations for venoms used in assays was 0.0037–5 µg/mL, demonstrating large differences among venoms in the proportion or relative sPLA2 activity for this substrate. For 13 elapid venoms final concentrations ranged from 0.0037 µg/mL for *M. fulvius* to 100 µg/mL for *Dendroaspis polylepis* (mean concentration 7.8 ± 28 µg/mL; median concentration 0.1 µg/mL) and for 15 viper venoms a range of 0.033 µg/mL, e.g., *V. berus* and 25 µg/mL *Calloselasma rhodostoma* (mean concentration 2.47 ± 6.35 µg/mL; median concentration 1 µg/mL). Instrumentation used included Tecan Aquarius (Männedorf, Switzerland), Matrix PlateMatePlus (Hudson, NH, USA), Titertek (Pforzheim, Germany) and Thermo (Hudson, NH, USA) Multidrop liquid dispensers and Tecan infinite M1000 (Männedorf, Switzerland) plate readers. Piloting collections used in the first phases of screening were selected by the inventor or from libraries of known compounds and natural products available at the time of experiments on selected venoms including: NIH Clinical Collection, GenPlus, Pharmakon, Bioactive lipids, Protease Inhibitors, Procured Drugs and FDA Approved Drugs libraries. The GenPlus (the NINDS Custom Collection) from MicroSource Discovery Systems contains 960 compounds. Varespladib outperformed all compounds in the library confirming (within the limits of the screening libraries) its superior potency as an sPLA2 inhibitor. No molecule in these collections totaling more than 4000 distinct chemical entities was found to be within two orders of magnitude potency of varespladib and results are not within the scope of this manuscript [43]. For inhibitor and dose-response testing, 10 µL of snake venom or bee venom (+control) was added to assay plates using a multichannel pipetman (Matrix, Hudson, NH, USA) or a multidrop dispenser (Thermo, Hudson, NH, USA or Titertek, Pforzheim, Germany). Compounds from the chemical libraries or prepared serial dilution master plates dissolved in DMSO were added to assay plates using a pin tool (V & P Scientific, Inc., San Diego, CA, USA) to transfer 20 nL of compounds. Final DMSO concentrations in the assay are 0.1%. Substrate was then added in 10 µL for a final assay volume of 20 µL. Controls populations were included on each plate in replicate wells. The negative control wells were vehicle (DMSO-only) with no small molecule compound. The positive control to simulate full venom activity inhibition were wells in which no venom was added, and assay buffer added in its place. Assay signals were measured at initiation and after 60 min of reaction time at room temperature. Signals were quantified on the Tecan infiniTe M1000 plate reader measuring absorbance at 405 nm. Signals at initiation were subtracted from the signals at 60 min. These background-corrected values were normalized to the mean of replicate negative and positive control wells within the plate. To define the normalization scale, the mean of the negative control well signals, representing full venom activity, was normalized to 100% effect and the mean of positive control well signals, representing complete inhibition of venom activity, was normalized to 0% effect. And wells within the plate were scaled accordingly. These calculations were performed in MicroSoft Excel. Data were transferred to GraphPad Prism (6th edition, 2014, La Jolla, CA, USA) plotted and fit to models, such that IC_50_ or EC_50_ values could be determined. Tests of significance were calculated by Student’s *t* and all others were descriptive.

### 5.2. Animal in Vivo Studies

*M. fulvius* and *V. berus* venoms had the highest sPLA2 activity in vivo and and after successful pilot survival studies was chosen for the Non-GLP study in rats and *V. berus* for mouse studies. Confirmatory studies were conducted at Pacific BioLabs (Hercules, CA, USA) and complied with all applicable sections of the Animal Welfare Act regulations, the Public Health Service Policy on Humane Care and Use of Laboratory Animals, and the Guide for the Care and Use of Laboratory Animals. Procedures used were reviewed and approved by the Pacific BioLabs Institutional Animal Care and Use Committee (IACUC) in compliance with the Animal Welfare Act. The animal protocol is: IACUC protocol (AN109690-01, 21 October 2015). CD-1 mice (Charles River Laboratories) and Sprague-Dawley Rats with implanted jugular venous catheters were supplied by Charles River Laboratories. 18 Sprague-Dawley rats weighing between 183 and 214 g at the time of the study had jugular vein cannulas surgically implanted by the supplier. Rats were randomly assigned to six treatment groups (*n* = 3 each) and received snake venom with and without varespladib. Experiments were performed at a Pacific Biolabs (Hercules, CA, USA) so the investigators did not conduct the experiments and were blinded as such. Animals were monitored for signs of toxicity for approximately 24 h. Blood samples (without anticoagulant) were collected from each rat prior to dose administration and post dose administration at approximately 30 min, 1 h and 4 h. Per protocol, nominal blood collection times were pre-dose administration and post-varespladib administration at 30 min ± 1 min., 1 h ± 1 min., and 4 h ± 5 min. There were no deviations from these specifications except for animals that died prior to the last scheduled blood collect ion at 4 h. Blood was processed to serum and analyzed by the AILAC certified contract research organization (Pacific Biolabs) to determine sPLA2 activity using Abcam kit (catalog number ab133089) validated beforehand with rat serum for quality control. Surviving animals were euthanized following the 24-h observation. Tissues were grossly examined but not collected for further processing. Justification for the use of the mouse in this study is based on the premise that animal testing is an appropriate and ethical prerequisite to testing new drugs in humans, and that data obtained from nonclinical animal models will have relevance to the behavior of the test material in humans. Because of the complex interactions that occur in vivo, an in vitro system does not provide sufficient information for evaluation of a compound’s in vivo activities. It was expected that the number of animals used in this study would provide a large enough sample for scientifically meaningful results while using the fewest possible animals to achieve that result. The intravenous route was chosen to maximize the bioavailability of varespladib. All experiments were designed to insure 100% mortality in control animals within the expected ½ life of the test drug in order to produce clear results using the lowest number of animal.

## Figures and Tables

**Table 1:** Varespladib and methyl-varespladib have breadth and potency against 28 medically important venoms from six continents (Vipers *n* = 15, Elapids *n* = 13) in vitro (Common English names are in parentheses). IC_50_ (µM) were calculated using chromogenic assays for sPLA2 inhibition; *R*-square for dose response curves 0.96 ± 0.04 (95% C.I. 0.94–0.98). While demonstrating high degrees of potency against snake venoms, neither varespladib nor methyl-varespladib showed high degrees of potency against bee venom sPLA2 (positive control).

Venom	Geographic Range	Varespladib IC_50_ µM	Me-Varespladib IC_50_ µM
Bee Venom	Worldwide	13.25	* Indeterminate
*Acanthophis antarcticus* (Common death adder)	Australia/PNG	0.0006	Not tested
*Agkistrodon blomhoffii brevicaudus* (Mamushi)	SE Asia, Japan	0.0005	0.04
*Agkistrodon contortrix* (Copperhead)	N. America	0.0002	Not tested
*Agkistrodon piscivorus* (Cottonmouth)	N. America	0.0003	Not tested
*Bitis gabonica* (Gaboon viper)	Africa	0.0003	Not tested
*Bothrops asper* (Fer-de-lance)	S. America	0.0001	Not tested
*Bothrops jararaca* (Jararaca)	S. America	0.0002	Not tested
*Bungarus caeruleus* (Common krait)	India/Asia	0.0001	0.02
*Bungarus fasciatus* (Banded krait)	India/Asia	0.00003	0.01
*Calloselasma rhodostoma* (Malayan pit viper)	SE Asia	0.002	Not tested
*Crotalus adamanteus* (Eastern diamondback rattlesnake)	N. America	0.0002	0.02
*Crotalus atrox* (Western diamondback rattlesnake)	N. America	0.0003	0.04
*Crotalus durissus terrificus* (South American rattlesnake)	S. America	0.005	0.26
*Crotalus scutulatus scutulatus* (Mojave green rattlesnake)	N. America	0.002	0.21
*Dendroaspis polylepis* (Black mamba)	Africa	0.00003	0.02
*Echis carinatus* (Saw-scaled viper)	India/Pakistan	0.00006	0.009
*Laticauda semifasciata* (Banded sea krait)	Pacific Ocean	0.00006	0.02
*Micrurus fulvius* (Eastern coral snake)	N. America	0.001	0.08
*Naja naja atra* (Chinese cobra)	China/Taiwan	0.0008	0.01
*Naja naja kaouthia* (Monocled cobra)	India/Asia	0.00005	0.02
*Naja naja naja* (Spectacled or Indian cobra)	India	0.001	0.02
*Notechis scutatus scutatus* (Tiger snake)	Australia	0.00006	0.03
*Ophiophagus hannah* (King cobra)	India/Asia	0.003	0.001
*Oxyuranus scutellatus* (Coastal taipan)	Australia/PNG	0.001	0.01
*Pseudechis australis* (Mulga snake)	Australia	0.003	0.09
*Trimersurus elegans* (Elegant pit viper)	SE Asia	0.0007	Not tested
*Vipera berus* (Common European adder)	Europe/Asia	0.00002	0.03
*Vipera russelli* (Russell’s viper)	India/Asia	0.0006	0.02

* Indeterminate = No apparent effect. PNG, Papua New Guinea, N., North, S., South, SE, South East.

## Abbreviations

The following abbreviations are used in this manuscript:

svMP	Snake venom metalloprotease
SC	Subcutaneous
PLA2	Phospholipase A2
sPLA2	secretory Phospholipase A2
